# Comparative analysis of early immune responses induced by two strains of Newcastle disease virus in chickens

**DOI:** 10.1002/mbo3.701

**Published:** 2018-08-01

**Authors:** Tingting Zhang, Mengting Ren, Chenggang Liu, Liwen Xu, Fangfang Wang, Zongxi Han, Yuhao Shao, Deying Ma

**Affiliations:** ^1^ College of Animal Science and Technology Northeast Agricultural University Harbin China; ^2^ Division of Avian Infectious Diseases State Key Laboratory of Veterinary Biotechnology Harbin Veterinary Research Institute Chinese Academy of Agricultural Sciences Harbin China

**Keywords:** chicken, immune response, Newcastle disease virus, viral RNA

## Abstract

Newcastle disease, caused by virulent strain of Newcastle disease virus (NDV), is an acute, highly contagious disease that is prevalent worldwide and is responsible for serious economic losses to the poultry industry. In the current study, we compared the early immune responses in chickens infected with two strains of velogenic NDV, a duck origin, named GD strain (Md/CH/LGD/1/2005, genotype VIId), and an chicken origin, F48E9 strain (genotype IX). The viral RNA level of GD strain was significantly higher than those of F48E9 in most tissues of chicken. Furthermore, the high level of viral RNA of the GD strain was associated with a stronger immune response compared to that of F48E9, characterized by upregulated expression of some of avian β‐defensins and cytokines, most of toll‐like receptors, and some of the other immune‐related genes investigated. This study thus demonstrated differences in host early immune responses to the two NDV strains. Further studies are needed to characterize the basic molecular mechanisms involved in the host responses in chickens infected by the two NDV strains.

## 1 INTRODUCTION

Newcastle disease (ND) is one of the most important viral diseases affecting birds worldwide, and is responsible for serious economic losses to the poultry industry due to high mortality, decreased egg production, and body weight loss (Alexander, 1997). The causative agent, virulent ND virus (NDV), belongs to genus *Avulavirus* of the *Paramyxoviridae* family. Virulent NDV continues to be endemic in many countries, despite the application of vaccines (Dimitrov, Afonso, Yu, & Miller, 2016). NDV can infect a wide variety of birds, but chickens remain the most important and susceptible host (Dimitrov, Ramey, Qiu, Bahl, & Afonso, 2016). Genotype VII is the most prevalent NDVs found in China in recent years, and has been considered to be mainly responsible for outbreaks of ND in this country (Liu, Wan, Ni, Wu, & Liu, 2003; Qin et al., 2008; Yu, Wang, Jiang, Chang, & Kwang, 2001).

Despite the importance of the antibody‐mediated response for protecting against NDV infection, the innate immune response induced by NDV challenge remains unclear. The host innate immune system provides the first line of defense against pathogens. The innate immune system can recognize components of pathogens called pathogen‐associated molecular patterns via pattern recognition receptors (Amimo et al., 2014; Yan et al., 2017). Toll‐like receptors (TLRs) are a family that belongs to the pattern recognition receptors. So far, approximately 13 TLRs have been identified in mammals (Paul, Brisbin, Abdul‐Csreem, & Sharif, 2013) and 10 TLRs have been identified in avian species (Brownmlie & Allan, 2011). Once the pathogen‐associated molecular patterns are recognized by TLRs, the TLRs may then trigger downstream signal transduction through activation of MyD88‐dependent or ‐independent intracellular pathways, to activate NF‐κB transcription factors and mitogen‐activated protein kinase to induce immune‐related molecules, such as host defense peptides and cytokines to defend against the invading pathogens (Akira & Takeda, 2004; Seth, Sun, & Chen, 2006; Takeuchi & Akira, 2010). In the past several years, innate immune responses to different strain of virus infections have been studied. Different expression levels of TLRs, cytokines, avian β‐defensins (AvBDs) have been detected in tissues following challenge of chickens, geese, and pigeon with different strains of NDV (Abdel‐Mageed, Isobe, & Yoshimura, 2014; Li et al., 2015; Xu et al., 2016; Yan et al., 2017). In addition, innate immune response to some other avian virus infection have been also reported, such as innate immunity of duck against duck hepatitis virus (Ma et al., 2011, 2012), innate immunity of chicken against infectious bronchitis virus (Xu et al., 2015).

Although numerous studies have focused on the pathogenesis of different isolates and pathotypes of NDV, limited data are available regarding the host immune responses induced by different NDV strains. A recent study demonstrated that infection with a NDV strain IBS002 (genotype VII) caused an earlier cytokine peak and higher viral load compared to those of AF2240 strain (genotype VIII) (Rasoli et al., 2014). Furthermore, a NDV strain JS5/05 (genotype VIId) produced more severe damage to the lymphatic organs in chickens compared to Herts/33 strain (genotype IV) and F48E8 strain, due to the high viral load and strong immune response induced by JS5/05 strain (Hu et al., 2015). These findings indicated that differential regulation of the host immune response by different NDV strains is an important aspect of NDV pathogenesis.

In this study, we evaluated the molecular bases of the different immune responses induced by two NDV strains, GD (a genotype VIId strain,isolated from the kidney of a diseased white Muscovy duck during an outbreak in 2005) and F48E9 by measuring the viral RNA and expression patterns of avian β‐defensins (AvBDs), TLRs, cytokines, and other immune‐related genes in the tissue samples of chickens during the early stage of infection.

## 2 MATERIALS AND METHODS

### 2.1 Virus strains

The velogenic NDV genotype VII strain GD (Md/CH/LGD/1/2005) was isolated from kidney samples from a diseased white Muscovy duck during an outbreak in 2005 (Wu et al., 2015), and the virulent NDV genotype IX strain F48E9 was isolated from chickens and stored at the Laboratory of Avian Infectious Diseases, State Key Laboratory of Veterinary Biotechnology, Harbin Veterinary Research Institute (HVRI), Chinese Academy of Agricultural Sciences (Harbin, China). According to pathogenicity tests in chickens, the GD strain had an intracerebral pathogenicity index of 1.88 and a mean death time of 50 hr, compared to 1.63 and 71 hr, respectively, in ducks (Wu et al., 2015). The intracerebral pathogenicity index for the F48E9 strain in chickens was 2.0 (Guo et al., 2014). Viruses were purified and propagated as described by Xu et al. (2016). Infective allantoic fluid containing virus was harvested and stored at −70°C until use.

All the experiments involving viruses were conducted in a purifier class II biosafety cabinet in a biosafety level‐2 laboratory.

### 2.2 Animals

One‐day‐old specific‐pathogen‐free chickens were obtained from the Laboratory Animal Center, HVRI. The chickens were maintained in isolators with negative pressure, and food and water were provided ad libitum until use. All these birds were negative for NDV antibody.

### 2.3 Preparation of standards for real‐time reverse transcription‐polymerase chain reaction (rRT‐PCR)

We obtained standard DNA for quantification of target gene transcripts, including 18S rRNA, TLRs (1, 2, 3, 4, 5, 7, 15 and 21), AvBDs (1–14), inducible nitric oxide synthase (iNOS), MHC class I and II, cytokines (interleukin (IL)‐1β, IL‐2, IL‐6, IL‐8, and IL‐18, and interferon‐γ (IFN‐γ), Fas/Fas ligand (FasL), myeloid differentiation primary response 88 (MyD88), interferon regulatory factor (IRF)‐7, nuclear factor‐κB (NF‐κB), and the M gene from both NDV strains, by RT‐PCR amplification with specific primers (Supporting Information Table S1). Total RNA was extracted from bone marrow or spleen from healthy chickens, and cDNA was synthesized as described by Li et al. (2015). The plasmid products were prepared as described previously (Li et al., 2015), and used as respective standards for subsequent quantitative RT‐PCR.

### 2.4 Experimental design and quantitative RT‐PCR of mRNAs in tissues

Chickens at 20 days of age were allotted randomly to three groups of ten each. Groups 1 and 2 were inoculated intranasally with 100 μl of the GD strain at 10^6.2^ 50% egg infective doses (EID_50_), or the same dose of F48E9 respectively. Group 3 was inoculated with 100 μl of phosphate‐buffered saline served as a control. Five birds from each group were slaughtered at 24 and 48 hr postinfection (hpi) respectively. Tissue samples of lung, trachea, proventriculus, kidney, cecal tonsil, liver, spleen, Harderian gland, bone marrow, and brain were collected and used for quantitative RT‐PCR analyses of host genes and NDV. The mRNA expression levels of the target genes were measured using One‐step Real‐time PrimeScript RT‐PCR (Takara Biotechnology, Dalian, China) with specific primers (Supporting Information Table S1), as described previously (Li et al., 2015; Xu et al., 2016). All amplifications were performed in triplicate. The mRNA levels of each target gene were normalized to the levels of 18S rRNA in the same samples.

### 2.5 Statistical analyses

Data are expressed as the mean ± *SD*. The significance of the results was assessed using SPSS, version 18 (SPSS, Armonk, NY, USA). *p* < 0.05 was considered to be statistically significant.

## 3 RESULTS

### 3.1 Different viral RNA levels in tissues of chickens infected with GD and F48E9 strains

**Figure 1:**
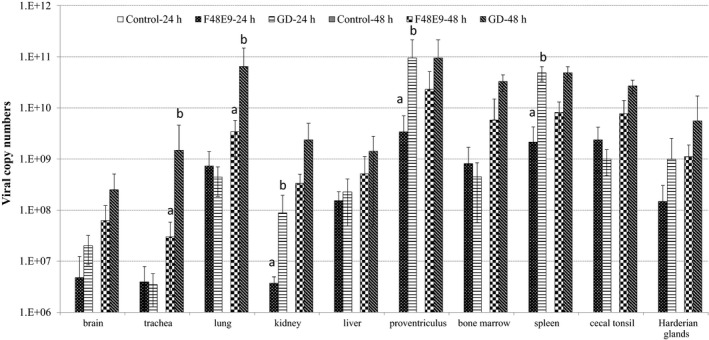
Viral replication in tissues of chickens challenged with Newcastle disease virus (NDV) strain GD (Md/CH/LGD/1/2005) and NDV strain F48E9, respectively. Viral RNA copy numbers in tissue samples were measured by quantitative RT‐PCR. Each bar indicates the mean ± *SD*. ^a–c^Values with different superscripted letters are significantly different (*p* < 0.05)

We investigated the differences in virus distribution in tissues of chickens infected with the two different NDV strains at 24 and 48 hpi. Both NDV strains successfully established an early infection, and viral RNA was detected in all the tested tissues at 24 and 48 hpi. In contrast, no viral RNA was detected in tissues from control birds. Of these tissues investigated, GD viral RNA level were significantly higher in the kidney, proventriculus, and spleen at 24 hpi, and in the trachea and lung at 48 hpi, than those of F48E9 (*p* < 0.05) (Figure 1).

### 3.2 GD infection upregulated expression of AvBDs in chicken tissues

**Figure 2:**
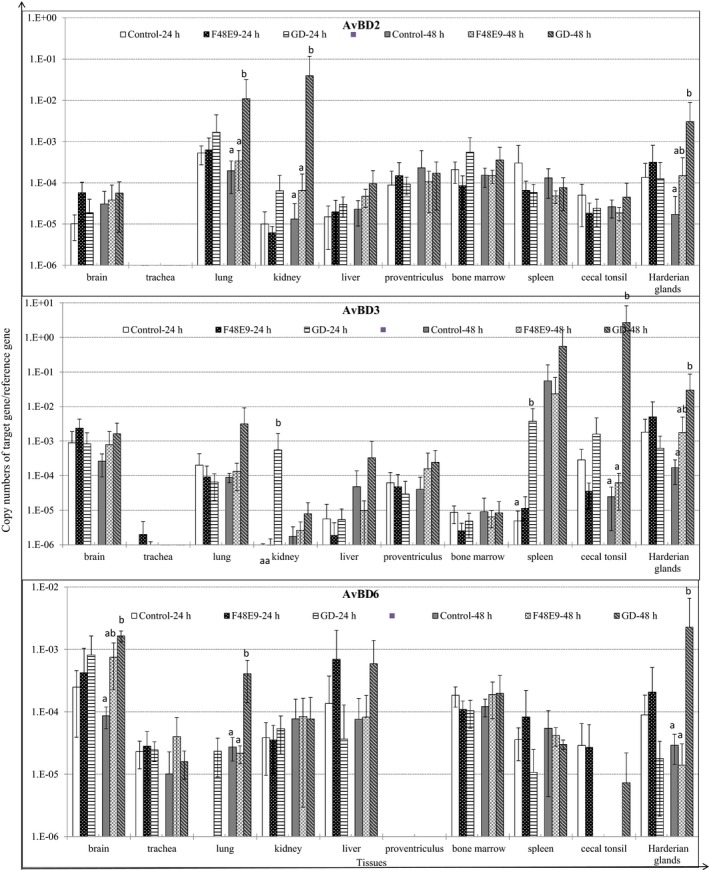
Relative gene expression levels of AvBDs in tissues of chickens challenged with Newcastle disease virus (NDV) strain GD (Md/CH/LGD/1/2005) and NDV strain F48E9 respectively. The cDNA copy numbers in the tissue samples were measured by quantitative RT‐PCR. The mRNA levels of target genes were normalized to that of 18S rRNA in the same samples. Each bar is the mean ± *SD*. ^a,b^Values with different superscripted letters are significantly different (*p* < 0.05)

The expression patterns of AvBDs 1–13 were investigated in chickens infected with the two NDV strains respectively. Of these AvBDs investigated, GD infection significantly upregulated AvBD2 in lung, kidney, and Harderian glands at 48 hpi, AvBD3 in kidney and spleen at 24 hpi, in cecal tonsil and Harderian glands at 48 hpi, and AvBD6 in brain, Harderian glands, and lung at 48 hpi, as compared to those of the control and F48E9 (*p* < 0.05) (Figure 2). In contrast, neither of NDV strains infection had obviously effects on the expression levels of the other AvBDs (data not shown).

### 3.3 GD triggered stronger cytokine response than F48E9

**Figure 3:**
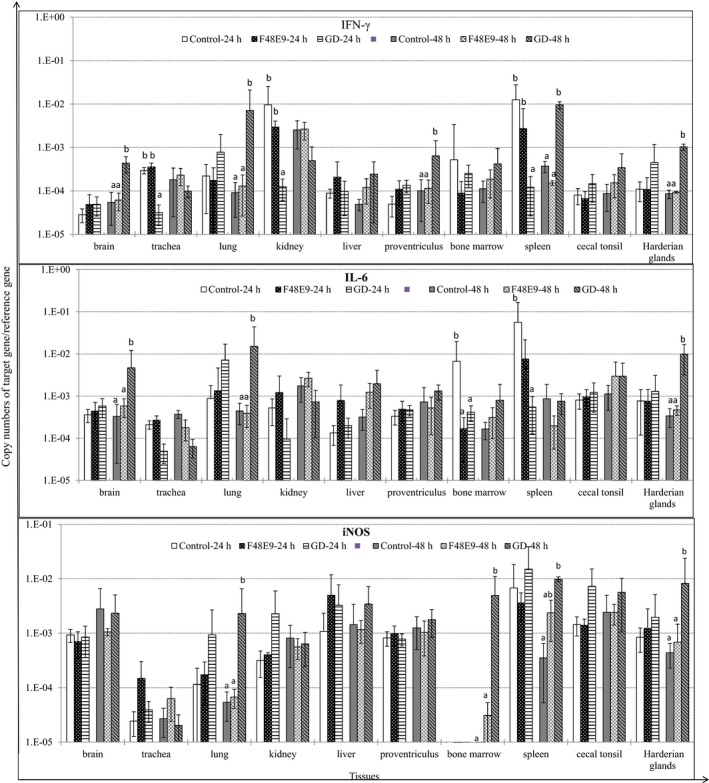
Relative gene expression levels of cytokines and inducible nitric oxide synthase (iNOS) in tissues of chicken challenged with Newcastle disease virus (NDV) strain GD (Md/CH/LGD/1/2005) and NDV strain F48E9, respectively. cDNA copy numbers in the tissue samples were measured by quantitative RT‐PCR. The mRNA levels of target genes were normalized to that of 18S rRNA in the same samples. Each bar is the mean ± *SD*. ^a,b^Values with different superscripted letters are significantly different (*p* < 0.05)

GD infection inhibited IFN‐γ expression in the trachea, kidney, and spleen at 24 hpi (*p* < 0.05) (Figure 3). In contrast, GD infection significantly upregulated expression levels of IFN‐γ in the brain, lung, proventriculus, spleen, and Harderian glands at 48 hpi, compared to the control (*p* < 0.05). Both strains of NDV decreased IL‐6 mRNA expression in the bone marrow and spleen at 24 hpi (*p* < 0.05). The IL‐6 expression was remarkably upregulated in the brain, lung, and Harderian glands at 48 hpi following GD infection (*p* < 0.05). However, neither of NDV strains had any significant effect on mRNA expression levels of IL‐1β, IL‐2, IL‐8, and IL‐18 in any of the tested tissues (data not shown). Furthermore, GD infection significantly upregulated iNOS expression in the lung, bone marrow, and Harderian glands at 48 hpi, compared to the control and F48E9, and spleen at 48 hpi, compared to the control (*p* < 0.05) (Figure 3). Overall, these data suggested that GD infection triggered a more potent cytokine response than infection with the F48E9 strain.

### 3.4 GD infection trigged strong TLR responses in chicken tissues

**Figure 4:**
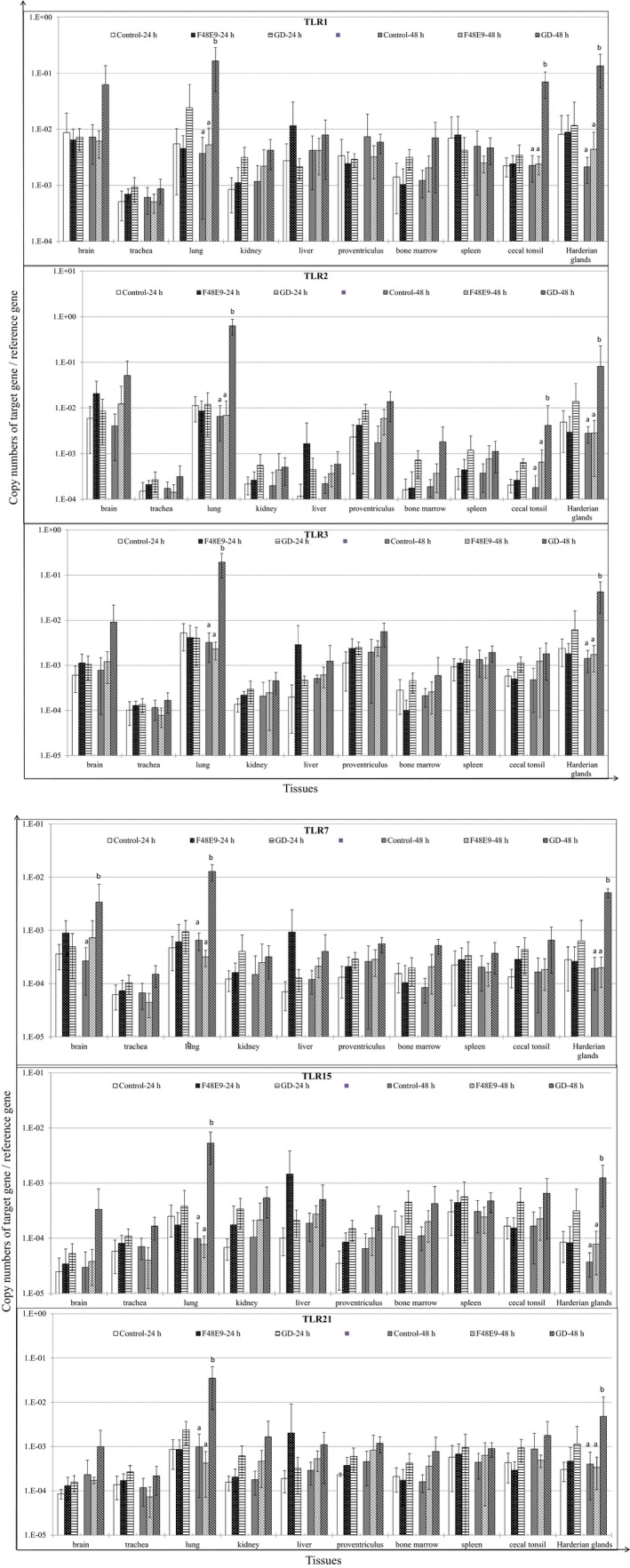
Relative gene expression levels of toll‐like receptors (TLRs) in tissues of chickens challenged with Newcastle disease virus (NDV) strain GD (Md/CH/LGD/1/2005,) and NDV strain F48E9 respectively. The cDNA copy numbers in the tissue samples were measured by quantitative RT‐PCR. The mRNA levels of target genes were normalized to that of 18S rRNA in the same samples. Each bar is the mean ± *SD*. ^a,b^Values with superscripted different letters are significantly different (*p* < 0.05)

GD infection significantly upregulated the expression of most TLRs (except TLR4 and TLR5) in both Harderian glands and lung, and TLR1 and TLR2 in cecal tonsil at 48 hpi (*p* < 0.05), compared to the control and F48E9 (Figure 4). In contrast, gene expression levels of TLR4 and TLR5 in all the tested tissues were unaffected by either NDV strains infection (data not shown).

### 3.5 GD infection trigged the other immune‐related molecular responses in chicken tissues

Of the immune‐related molecules investigated, MyD88 mRNA expression was significantly upregulated in bone marrow at 24 hpi and in Harderian gland at both time points following infection with both NDVstrains (*p* < 0.05) (Supporting Information Figure S1). NF‐κB 52 was significantly upregulated in spleen at 24 hpi and in Harderian glands at both time points, and NF‐κB 65 was significantly upregulated in bone marrow at 48 hpi (*p* < 0.05) in response to GD infection, while none of the isoforms were significantly affected by F48E9 infection (Supporting Information Figure S2).

GD infection also greatly increased the expression of FasL in bone marrow, Fas in lung and Harderian glands, and MHC class I in brain, lung, and Harderian glands at 48 hpi (*p* < 0.05). Notably, MHC class II expression in the kidney was downregulated by GD infection at both 24 and 48 hpi and in spleen at 48 hpi (*p* < 0.05) (Supporting Information Figure S3). These results suggested that these molecules might involve in the innate immune response of chickens to GD infection.

## 4 DISCUSSION

Domestic waterfowl and chickens are traditionally raised together in most areas of China. However, this farming pattern may facilitate the transmission of various viruses, including NDV, among these domestic birds. Numerous studies have investigated the pathogenicity of NDV isolates, but there is currently limited information regarding the antiviral immune response in birds.

In this study, we evaluated the immune responses induced in chickens by two NDV strains, GD and F48E9. Despite lower ICPI, GD induces stronger immune response, as well as higher viral RNA level in tissues at early stage of infection, compared to that of F48E9 in this study. In consistent with this observation, it was previously reported that two NDV strains, JS3/05 and JS5/05 (ICPI 1.88) replicated more efficiently, and induced stronger immune responses in splenocytes of chicken at 6, 12, and 24 hpi in vitro (Hu et al., 2012) or in spleen, thymus, and bursa tissues of chicken at 24, 48, and 72 hpi in vivo (Hu et al., 2015), compared to that of F48E9 (ICPI 2.0). These observations revealed that the strong immune response caused by GD strain, as well as JS3/05 and JS5/05 strains, might be associated with high levels of viral load in infected tissues or cells at the early stage of infection. However, the underlying molecular mechanisms need to be further investigated. In addition, chickens and geese challenged by goose‐ or chicken‐origin (VIId genotypes) of NDV strains manifested similar pathological features, distinct from those of birds infected with Herts/33 strain (Wang et al., 2012). These results revealed that the viral load of NDV may be associated with the specific genotype, rather than with their origin.

Our results also indicated that GD infection upregulated the expression of three of all of AvBDs, compared to F48E9. Fourteen AvBDs have been identified in chickens to date, and are considered as key components of the host innate immune response against pathogens, and potentially provide a link between innate and adaptive immunities. In agreement with the present observations, expression levels of AvBDs were upregulated in tissues of various avian species infected with NDV strains of pigeon or goose origin (Li et al., 2015; Xu et al., 2016), as well as with other avian viruses (Ma et al., 2011, 2012; Xu et al., 2015). However, differences in their expression levels in chickens infected with different NDV strains have not previously been studied. Interestingly, we found that a higher viral load was associated with upregulation of these AvBDs in GD‐infected chickens, compared with F48E9 infection. These results revealed that the viral RNA level in tissues played a pivotal role in regulating expression of these AvBDs.

Considering their key roles in the regulation of immune responses, cytokine gene expression levels are used extensively as an indicator of the host immune response to virus infection. Surprisingly, the current study showed that both IFN‐γ and IL‐6 expression decreased in some tissues at 24 hpi following GD infection. This observation is different from most of previous findings (Abdel‐Mageed et al., 2014; Hu et al., 2012, 2015; Kapczynski, Afonso, & Miller, 2013). Moreover, the result showed that expression of IFN‐γ in trachea, kidney, and spleen, and IL‐6 expression in spleen was higher at 24 hpi following F48E9 infection than that of GD infection. This result is also different from their expression patterns at 48 hpi in most tissues. In order to verify this observation, several time experiments were repeated. Indeed, we could not figure out what factors cause this kind of special expression pattern, and what kind of roles both cytokines would play on NDV infection at this time point. Similarly, previous studies with Respiratory syncytial virus and Influenza virus demonstrated that viruses seem to suppress the production of type I IFNs by disrupting the signaling pathways of type I IFN synthesis (Oshansky, Krunkosky, Barber, Jones, & Tripp, 2009; Xia et al., 2016). However, whether the NDV strains have devised similar strategies to interfere with the production of IFN‐γ and IL‐6 at the time point of infection needed to be further elucidated.

In contrast, expression of IFN‐γ and IL‐6 in some tissues was increased at 48 hpi following both GD and F48E9 infection, compared to that of the control. These findings were partly in agreement with those of Hu et al. (2012), who showed that JS3/05 and JS5/05 NDV strains induced a more potent IFN response than F48E9 strain. In addition, our results, as well as those of Hu et al. (2012), also showed that the higher IFN‐γ expression induced by GD, JS3/05, or JS5/05 infection was associated with a high viral load, compared to F48E9. Cytokines could induce a specific immune response to prevent infection upon virus invasion. IFN‐γ mediates the production of nitric oxide, resulting in the recruitment of more neutrophils and macrophages (Huang et al., 2012). IL‐6 regulates immune and inflammatory responses involved in the activation, growth, and differentiation of T cells, and contributes to T‐cell‐mediated inflammatory reactions (Huang et al., 2012). However, overproduction of cytokines as a result of virus infection may result in a cytokine storm, thus amplifying the detrimental effect of inflammation on the host (Tisoncik et al., 2012). Notably, we showed that iNOS expression was upregulated in some tissues of chickens at 48 hr after GD infection. These results showed that high levels of cytokines induced by GD may be associated with the fasted virus replication, and then to further overcome the antiviral effects of these cytokines.

TLRs can respond to viral infection in both mammals and birds, and produce antiviral factors that help inhibit viral replication (Kapczynski et al., 2013; Li et al., 2015; Xu et al., 2016). Consistent with this observation, the present study showed that expression levels of most TLRs were significantly upregulated in some chicken tissues at 48 hr after infection with either GD or F48E9. Consistent with this finding, TLRs expression levels were induced in different tissues of NDV‐infected geese (Xu et al., 2016), while TLR3, TLR7, and TLR15 were induced in some tissues of pigeons infected with pigeon paramyxovirus type 1, a variant strains of NDV (Li et al., 2015). Overall, these results suggested that these TLRs may be involved in the host antiviral defense against both NDV strains.

In this study, the upregulation of MyD88, NF‐κB 52, and NF‐κB 65 in response to both GD and F48E9 infection was observed. Accordingly, previous reports showed that MyD88 and NF‐κB played important roles in innate antiviral immunity against West Nile virus (Daffis, Samuel, Keller, Gale, & Diamond, 2007; Daffis et al., 2008), dengue virus (Nasirudeen et al., 2011), avian Tembusu virus (Chen et al., 2016), and avian reticuloendotheliosis virus (Miao et al., 2015) infections. The current results suggested that these molecules may play similar roles in innate antiviral immunity against both strains of NDV. However, further studies are needed to determine whether other transcription factors are involved in innate immunity against NDV infection.

The apoptotic response has been identified as a key element in the pathogenesis of virus infection. FasL is strongly stimulated by the NF‐κB pathway in response to virus infections, such as H9N2 avian influenza virus infection (Xing, Cardona, Anunciacion, Adams, & Dao, 2010). Consistent with previous studies on other viruses, we showed that Fas/FasL was induced following GD infection, suggesting that Fas/FasL may be involved in the immune response to GD infection. In addition, MHC class I and class II were also upregulated in response to GD infection in this study. Consistent with this observation, MHC class I was upregulated y avian influenza H3N2 virus (Tong, Long, Li, & DeMaria, 2004) and by a NDV strain of goose origin (Xu et al., 2016). MHC molecules are critical components of the vertebrate adaptive immune system (Flajnik & Kasahara, 2010), and the current and previous results demonstrated critical roles for these molecules in the host defense against viruses.

## 5 CONCLUSION

We showed that the viral RNA of GD strain in most tissues of chickens was higher than those of F48E9 strain at the early stage of infection. The higher viral RNA level of the GD strain may be associated with a stronger innate immune response compared to the F48E9 strain. However, this study only analyzed differences in the host immune responses to infection with the two NDV strains at the early stage, and further investigations are needed to elucidate how these NDV strains induce differential host responses in chickens.

## CONFLICT OF INTEREST

The authors declare that they have no competing interests.

## ETHICS STATEMENT

All animal experiments were approved by the Ethical and Animal Welfare Committee of Heilongjiang Province, China (License no. SQ20150122).

## Data Availability

“The main data supporting this study are provided in the results section of this paper. The others data are provided as supplementary information accompanying this paper.”
