# Supplementary material for: Comparative analysis of early immune responses induced by two strains of Newcastle disease virus in chickens
Source: Microbiologyopen. 2018 Aug 1;8(4):e00701. doi: 10.1002/mbo3.701 (PMC6460277; doi:10.1002/mbo3.701)
Supplement: Supplementary file 1 [file MBO3-8-e00701-s001.pdf]

MicrobiologyOpen

Tingting Zhang [1,2†], Mengting Ren [1,2†],Chenggang Liu [1,2], Liwen Xu [1,2], Fangfang Wang [1,2], Zongxi Han [2], Yuhao Shao [2], Deying Ma [1*]
[1] College of Animal Science and Technology, Northeast Agricultural University, Harbin 150030, PR China.
[2] Division of Avian Infectious Diseases, State Key Laboratory of Veterinary Biotechnology, Harbin Veterinary Research Institute, Chinese Academy of Agricultural Sciences, Harbin 150001, PR China.

[†] Tingting Zhang and Mengting Ren are joint first author.

[*] Corresponding author
Deying Ma
E-mail: madeying@neau.edu.cn

Figure S1. Relative gene expression levels of MyD88 and IRF-7 in tissues of chickens challenged with Newcastle disease virus (NDV) strain GD (Md/CH/LGD/1/2005) and NDV strain F48E9, respectively. The cDNA copy numbers in tissue samples were measured by quantitative RT-PCR. The mRNA expression levels of target genes were normalized to that of 18S rRNA in the same samples. Each bar is the mean ± SD. $^{a, b}$ Values with different superscripted letters are significantly different (P<0.05).

Figure S2. Relative gene expression levels of NF-κB in tissues of chickens challenged with Newcastle disease virus (NDV) genotype VIId strain GD (Md/CH/LGD/1/2005) and NDV strain F48E9, respectively. The cDNA copy numbers in tissue samples were measured by quantitative RT-PCR. The mRNA expression levels of target genes were normalized to that of 18S rRNA in the same samples. Each bar is the mean ± SD. $^{a, b}$ Values with different superscripted letters are significantly different (P<0.05).

Figure S3. Relative gene expression levels of Fas/FasL, MHC class I, and MHC class II in tissues of chickens challenged with Newcastle disease virus (NDV) strain GD (Md/CH/LGD/1/2005) and NDV strain F48E9, respectively. The cDNA copy numbers in tissue samples were measured by quantitative RT-PCR. The mRNA expression levels of target genes were normalized to that of 18S rRNA in the same samples. Each bar is the mean ± SD. $^{a, b}$ Values with different superscripted letters are significantly different (P<0.05).

Fig.S1

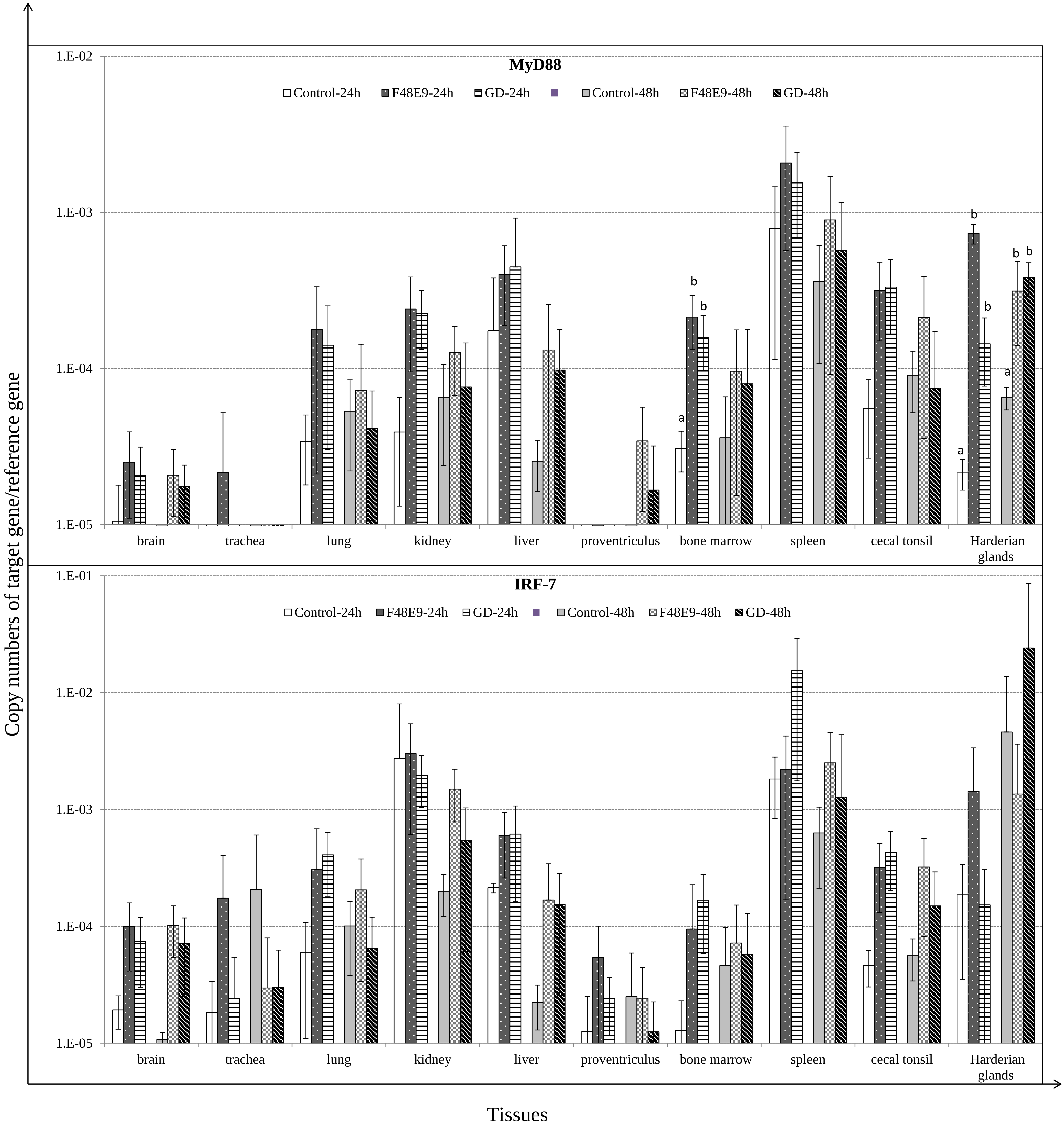
MyD88
Control-24h
F48E9-24h
GD-24h
Control-48h
F48E9-48h
GD-48h
1.E-02
1.E-03
1.E-04
1.E-05
a
b
b
a
b
b
a
b
b
brain
trachea
lung
kidney
liver
proventriculus
bone marrow
spleen
cecal tonsil
Harderian glands
IRF-7
Control-24h
F48E9-24h
GD-24h
Control-48h
F48E9-48h
GD-48h
1.E-01
1.E-02
1.E-03
1.E-04
1.E-05
brain
trachea
lung
kidney
liver
proventriculus
bone marrow
spleen
cecal tonsil
Harderian glands
Copy numbers of target gene/reference gene
Tissues

Fig.S2

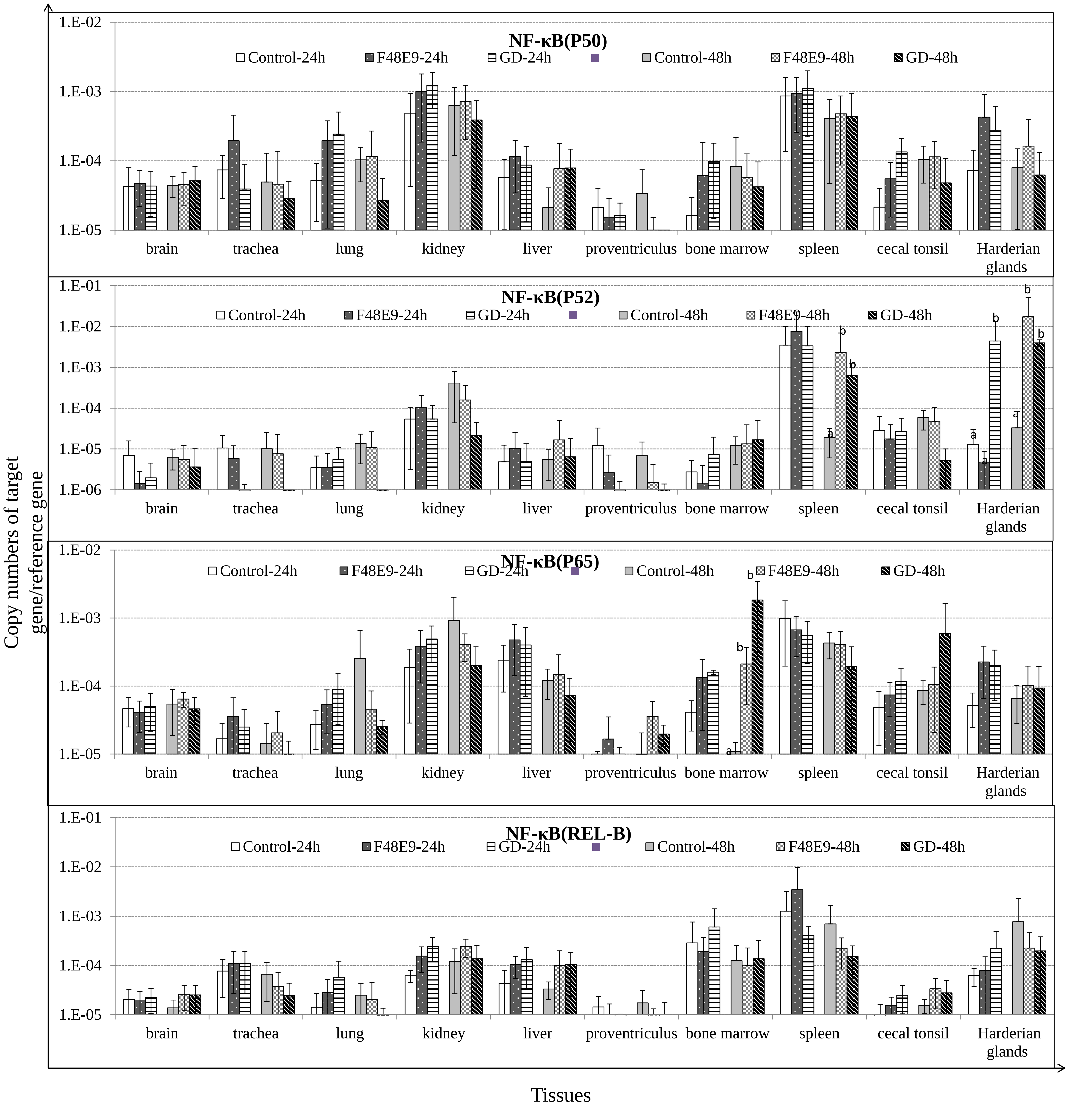
NF-κB(P50)
Control-24h
F48E9-24h
GD-24h
Control-48h
F48E9-48h
GD-48h
NF-κB(P52)
NF-κB(P65)
NF-κB(REL-B)
brain
trachea
lung
kidney
liver
proventriculus
bone marrow
spleen
cecal tonsil
Harderian glands
Copy numbers of target gene/reference gene
Tissues

Fig.S3

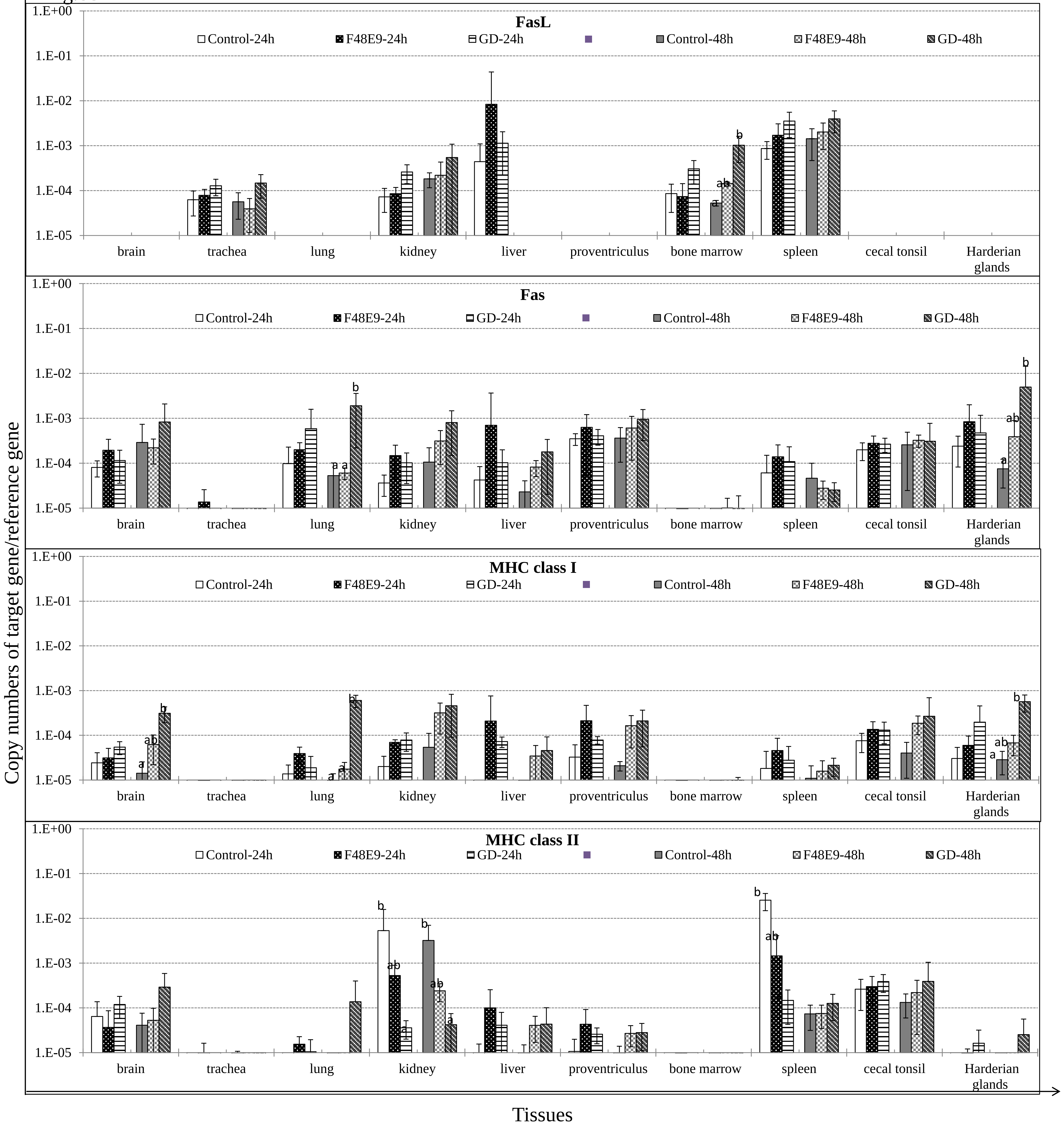

FasL
Fas
MHC class I
MHC class II
Control-24h
F48E9-24h
GD-24h
Control-48h
F48E9-48h
GD-48h
1.E+00
1.E-01
1.E-02
1.E-03
1.E-04
1.E-05
brain
trachea
lung
kidney
liver
proventriculus
bone marrow
spleen
cecal tonsil
Harderian glands
Copy numbers of target gene/reference gene
Tissues
