# Supplementary material for: Comparative analysis of early immune responses induced by two strains of Newcastle disease virus in chickens
Source: Microbiologyopen. 2018 Aug 1;8(4):e00701. doi: 10.1002/mbo3.701 (PMC6460277; doi:10.1002/mbo3.701)
Supplement: Supplementary file 2 [file MBO3-8-e00701-s002.docx]

**[MicrobiologyOpen](https://www.baidu.com/link?url=J-FBgpJ81-Qhw9VRg84EaswVMoenOFsdTcI9An5CBtN0IWQbLFQC0KZZklP7lpZg&wd=&eqid=f055761b0000ffe0000000025ab1bd93)**

Tingting Zhang ^1,2†^, Mengting Ren ^1,2†^,Chenggang Liu ^1,2^, Liwen Xu ^1,2^, Fangfang Wang ^1,2^, Zongxi Han ^2^, Yuhao Shao ^2^, Deying Ma ^1*^

^1^ College of Animal Science and Technology, Northeast Agricultural University, Harbin 150030, PR China.

^2^ Division of Avian Infectious Diseases, State Key Laboratory of Veterinary Biotechnology, Harbin Veterinary Research Institute, Chinese Academy of Agricultural Sciences, Harbin 150001, PR China.

^†^ Tingting Zhang and Mengting Ren are joint first author.

^*^ Corresponding author

Deying Ma

E-mail: [madeying@neau.edu.c](mailto:madeying@neau.edu.c)n

Table S1 Real time PCR primer sequences

| Target mRNA | Sense primer (5`-3`) | Antisense primer (5`-3`) | Product size (bp) | GenBank acession no. |
| --- | --- | --- | --- | --- |
| NDV | 5′-AGTGATGTGCTCGGACCTTC-3′ | 5′-CCTGAGGAGAGGCATTTGCTA-3′ | 121 | AF089819 |
| 18S rRNA | 5′-TCAGATACCGTCG TAGTTCC-3′ | 5′-TTCCGTCAATTCCTTTAAGTT-3′ | 154 | FM165414 |
| AvBD1 | 5′-GATCCTCCCAGGCTCTAGGAAG-3′ | 5′-GCCCCATATTCTTTTGC-3′ | 137 | NM_204993 |
| AvBD2 | 5′-GGTTGTCTTCGCCCCGGCGGGA -3′ | 5′-TTATGCATTCCAAGGCCATTTG-3′ | 137 | NM_204992 |
| AvBD3 | 5′-GAACTGCCACTCAGTGCAGAAT-3′ | 5′-ATGGGGGTTGTTTCCAGGAGC-3′ | 182 | NM_ 204650 |
| AvBD4 | 5′-TCATCGTGCTCCTCTTTGTG-3′ | 5′-AATACTTGGGACGGCATAGC -3′ | 153 | NM_001001610 |
| AvBD5 | 5′-GCTGTCCCTTGCTCGAGGATT-3′ | 5′-GGAATACCATCGGCTCCGGC-3′ | 139 | NM_001001608 |
| AvBD6 | 5′-GTCAGCCCTACTTTTCCAGC-3′ | 5′-GCCCACCTGTTCCTCACAC-3′ | 143 | NM_001001193 |
| AvBD7 | 5′-ACCTGCTGCTGTCTGTCCTC-3′ | 5′-TGCACAGCAAGAGCCTATTC-3′ | 173 | NM_001001194 |
| AvBD8 | 5′-TTCTCCTCACTGTGCTCCAA -3′ | 5′-AAGGCTCTGGTATGGAGGTG-3′ | 124 | NM_001001781 |
| AvBD9 | 5′-GCTTACAGCCAAGAAGACGCT-3′ | 5′-GGAGCTGGGTGCCCATTTGCA-3′ | 145 | NM_001001611 |
| AvBD10 | 5′-GGCTCAGCAGACCCACTTTTCC -3′ | 5′-CTGCGCCGGAATCTTGGCAC-3′ | 146 | NM_001001609 |
| AvBD11 | 5′-GGTCTCGGCTTGCCCAGAGAC-3′ | 5′-ATGGAAGTCTGATGTAGTGTC-3′ | 150 | NM_001001779 |
| AvBD12 | 5′-GGAACCTTTGTTTCGTGTTCA-3′ | 5′-GAGAATGACGGGTTCAAAGC-3′ | 155 | NM_001001607 |
| AvBD13 | 5′-GATCCTCCAGCTGCTCTTTG-3′ | 5′-AGTGGCCATGGTTGTTCCT-3′ | 104 | NM_001001780 |
| TLR1 | 5′-AGTCCATCTTTGTG TTGTCGCC-3′ | 5′-ATTGGCTCCAGCAA GATCAGG-3′ | 127 | JF823982 |
| TLR2 | 5′-GATTGTGGACAACATCATTGACTC-3′ | 5′-AGAGCTGCTTTCAAGTTTTCCC-3′ | 294 | JN544178 |
| TLR3 | 5′-Tcagtacatttgtaacaccccgcc-3′ | 5′-ggcgtcataatcaaacactcc-3′ | 256 | NM_001011691 |
| TLR4 | 5′-AGTCTGAAATTGCTGAGCTCAAAT-3′ | 5′-GCGACGTTAAGCCATGGAAG-3′ | 190 | NM_001030693 |
| TLR5 | 5′-CCTTGTGCTTTGAGGAACGAGA-3′ | 5′-CACCCATCTTTGAGAAACTGCC-3′ | 124 | NM_001024586 |
| TLR7 | 5′-ttctggcc acagatgtgacc-3′ | 5′-ccttcaactt ggcagtgcag-3′ | 219 | NM_001011688 |
| TLR15 | 5′-GTTCTCTCTCCCAGTTTGTAAATAGC-3′ | 5′-GTGGTTCATTGGTTGTTTTTAGGAC-3′ | 262 | NM_001037835 |
| TLR21 | 5′-TGCCCCTCCCACTGCTGTCCACT-3′ | 5′-AAAGGTGCCTTGACATCCT-3′ | 112 | AF089819 |
| IFN-β | 5′-GCTCTCACCACCACCTTCTC-3′ | 5′-GCTGCTTGCTTCTTGTCCTTG-3′ | 179 | NM_001024836 |
| IL-2 | 5′-CCCAGCAAACTCTGCAGTGTT-3′ | 5′-CCGGTGTGATTTAGACCCGTA-3′ | 148 | NM_204628 |
| IL-6 | 5′-AGATGGTGATAAATCCCGATGA-3′ | 5′-CGGTCTTCTCCATAAACGAAGT-3′ | 150 | HM179639 |
| IL-8 | 5′-ATGAACGGCAAGCTTGGAGCT-3′ | 5′-GCCATAAGTGCCTTTACGATCAG-3′ | 278 | GU119895 |
| IL-18 | 5′-GAAACGTCAATAGCCAGTTGC-3′ | 5′-TCCCATGCTCTTTCTCACAACA-3′ | 213 | FJ788637 |
| IFN-γ | 5′-ACACTGACAAGTCAAAGCCGCACA-3′ | 5′-AGTCGTTCATCGGGAGCTTGGC-3′ | 129 | GU119890 |
| MyD88 | 5′-TTACGAAGGAAGCAGCAG-3′ | 5′-TGACAGTAGCAGATGAAGG-3′ | MyD88 | NM_001030962 |
| IRF-7 | 5′-GAAAACACTCCCACAGACAG-3′ | 5′-ATCCTCAGGCAAGGCATC-3′ | IRF-7 | NM_205372 |
| NF-kB p50 | 5′-CAGAGGAAGAGGCAGAAG-3′ | 5′-CCATAGGAAGGATAACTGAAC-3′ | 131 | D13719 |
| NF-kB p52 | 5′-GTCCTGTCACCGTATTCC-3′ | 5′-TCCACCTCCTCCTTATCC-3′ | 106 | U00111 |
| NF-kB p65 | 5′-ATTTGGGCATTCAGTGTGTG-3′ | 5′-CGCTCAGGTCGTATTCGG-3′ | 123 | D13721 |
| NF-kB RelB | 5′-CCAATGCGTCAAGAAGAAG-3′ | 5′-CGTTCATATCCACCTCCTG-3′ | 113 | D13794 |
| FAS | 5′-AAAGCACTCGGTTTGGAGGTT-3′ | 5′-TGTTCACACCGAGAAGAATTGC-3′ | 101 | NM_001199487 |
| FASLG | 5′-TAACAGGAAACCCCACACAGC-3′ | 5′-CCGGAAGAGCACATTGGAGTA-3′ | 149 | NM_001031559 |
| MHC class I | 5′-AAGAAGGGGAAGGGCTACAA-3′ | 5′-AAGCAGTGCAGGCAAAGATT-3′ | 222 | HQ141386 |
| MHC class II | 5′-CTCGAGGTCATGATCAGCAA-3′ | 5′-TGTAAACGTCTCCCCTTTGG-3′ | 277 | HQ203729 |
